# A pilot study evaluating a support programme for parents of young people with suicidal behaviour

**DOI:** 10.1186/1753-2000-3-20

**Published:** 2009-07-15

**Authors:** Lorna Power, Sophia Morgan, Sinead Byrne, Carole Boylan, Andreé Carthy, Sinead Crowley, Carol Fitzpatrick, Suzanne Guerin

**Affiliations:** 1 Department of Child Psychiatry, The Children's University Hospital, Temple Street, Dublin, Ireland; 2 Department of Psychology, University College Dublin, Ireland

## Abstract

**Background:**

Deliberate self harm (DSH) is a major public health concern and has increased among young people in Ireland. While DSH is undoubtedly the result of interacting factors, studies have identified an association between DSH and family dysfunction as well as the protective role of positive family relationships. Following a focus group meeting held to identify the needs of parents and carers of young people with DSH, a support programme (SPACE) was developed. The aims of the current study are to evaluate the effectiveness of the SPACE programme in decreasing parental psychological distress, reducing parental report of young peoples' difficulties, increasing parental satisfaction and increasing parents' ratings of their own defined challenges and goals.

**Methods:**

Participants were recruited from a Mental Health Service within a paediatric hospital, Community Child and Adolescent Mental Health Teams and family support services. All services were located within the greater Dublin area in Ireland. Forty-six parents of children who had engaged in or expressed thoughts of self harm attended the programme and participated in the evaluation study. The programme ran once a week over an 8-week period and included topics such as information on self harm in young people, parenting adolescents, communication and parental self-care. Seventy percent (N = 32) of the original sample at Time 1 completed measures at Time 2 (directly following the programme) and 37% (N = 17) of the original sample at Time 1 completed them at Time 3 (6 months following the programme).

A repeated measures design was used to identify changes in parental wellbeing after attendance at the programme as well as changes in parental reports of their children's difficulties.

**Results:**

Participants had lower levels of psychological distress, increased parental satisfaction, lower ratings of their own defined challenges and higher ratings of their goals directly after the programme. These changes were maintained at 6-month follow up in the 37% of participants who could be followed up. Furthermore the young people who had engaged in or expressed thoughts of self harm had lower levels of difficulties, as reported by their parents, following the programme.

**Conclusion:**

These findings suggest that the SPACE programme is a promising development in supporting the parents of young people with suicidal behaviour. The programme may also reduce parental reports of their children's difficulties. Further evaluation using a randomized controlled trial is indicated.

## Background

Deliberate Self Harm (DSH) is a major public health concern [1] which has become increasingly more common among young people. The term DSH describes "an act with a nonfatal outcome in which an individual deliberately did one or more of the following: initiated behaviour (for example, self-cutting, jumping from a height), which they intended to cause self-harm; ingested a substance in excess of the prescribed or generally recognised therapeutic dose; ingested a recreational or illicit drug that was an act that the person regarded as self-harm; ingested a non-ingestible substance or object" [2].

In a recent study of 4,583 adolescents in Ireland, a lifetime history of DSH was reported by 9.1% of respondents [1]. This is in contrast to a large self report survey of schools in England where a lifetime history of DSH was reported by 13.2% of respondents [2]. The Irish study was consistent with previous findings in that DSH was more common among females (13.9%) than males (4.3%). The most common methods used were cutting (66%) and taking an overdose (35.2%). Of those who had harmed themselves, only a minority (11.3%) had attended hospital afterwards. This is in line with the self-report survey in England where it was found that only 12.6% of episodes of self harm resulted in hospital presentation [2].

DSH is a significant risk factor for suicide in that individuals who take overdoses or deliberately inflict injury on themselves and survive are at a particularly high risk of eventually dying by suicide [3]. In addition there is an association between DSH in young people and a range of poor psychosocial outcomes as adults. A recent study [4] details the early adult outcomes of 132 adolescents who had deliberately self-poisoned. Participants were compared with a matched control group who had never harmed themselves and were randomly selected from the waiting lists of primary care physicians. Results indicated that rates of psychopathology, in particular depression, were higher among those who had self-poisoned – rates of current mental disorders were 16% in the control group and 39% in the self-poisoning group. Furthermore the self-poisoning group also differed to controls on a number of other measures of social functioning and adversity. The self-poisoning group were more likely than controls to have experienced sexual abuse, disrupted education, left school early without qualifications and to have left home, cohabited and become parents at an earlier age. Considering these associations, it is imperative to develop effective means of identifying and managing self harm in young people.

An episode of DSH is likely to have a negative effect on the families of the individual involved. In a qualitative study aimed at investigating parents' experiences after an episode of DSH in young people, parents reported feeling very distressed, helpless and anxious about the possibility of future episodes [5]. These concerns were perpetuated by a perceived lack of support and information from some health professionals. This suggests that parental support and education should be an integral part of aftercare.

In a similar study, aimed at investigating parents' emotional and behavioural responses to adolescents' suicide attempts [6], 22 mothers and 12 fathers were assessed soon after the event using both open-ended and structured interviews. Mothers' reactions included an increase in sad, caring and anxious feelings with approximately half feeling hostile after the suicide attempt. However, few verbalized this hostility and many reported being careful about what they said following the suicide attempt. The authors suggest that intervention with parents should focus on normalising their feelings and responses as well as developing the family's communication skills with a focus on increasing positive feedback and reducing hostile or critical statements.

While DSH is undoubtedly the result of multiple interacting factors with no one causal factor [7], there has been much research to support an association between DSH and poor family functioning. In a study of 20 individuals who had engaged in DSH but who had no further episodes in the two years prior to the interview, participants recalled unpredictability in family life at the time of self harming [8]. They also reported having felt unsupported, not heard and that their story was of no importance to their family. Poor communication in particular has been found to be associated with DSH. In a quantitative study comparing 52 adolescents who had presented to Accident and Emergency departments following DSH with 52 hospital-based controls whom had been admitted to the hospital and had no psychiatric history or self harm, there was a strong association between the absence of a family confidant and adolescent self harm [9]. The authors suggested that poor communication within the family may lead the young person to feel socially isolated, their problems to appear insurmountable with DSH being perceived as their only option. Likewise, positive parental behaviours can serve to protect young people from DSH. In a study of 451 families, who were participating in a longitudinal research project examining rural families in the United States, the family processes that may lead to adolescent suicidality were investigated [10]. Structural equation modelling was used to examine the hypothesis that parents' behaviour would predict their adolescents' emotional distress and subsequent suicidal behaviour. Findings indicated that warm and communicative behaviours conveyed by mothers had a direct negative association with adolescents' reporting of suicidality. These findings again emphasize the importance of parental involvement in treating DSH and suggest that parents may benefit from support which would include learning how to develop and foster effective communication skills with their adolescent.

### Design of SPACE programme

Temple Street Children's University Hospital is a tertiary referral teaching paediatric hospital in the centre of Dublin, Ireland. Due to a marked increase in the number of children and adolescents presenting following DSH, a consultant-led DSH team was established in 2002. Between 2002 and October 2008, the team assessed 458 young people aged 16 years and under, following an episode of DSH. During this time, the team identified a strong need for a programme to support parents and carers of young people who have engaged in DSH.

The SPACE (Supporting Parents and Carers) programme was designed as a support programme for parents and carers of children who have engaged in DSH. According to the Report of the Expert Group on Mental Health Policy in Ireland – A Vision for Change [11], service users should become involved in every aspect of service development and delivery. Considering this, as well as international endorsement of service user involvement, a Focus Group Meeting was held in order to directly establish the needs of parents and carers of young people who have engaged in DSH [12]. Twenty-five participants attended the meeting of whom 15 were parents and ten were carers. Participants were divided into subgroups and presented with two open ended questions a) What areas do you think a support group should address? b) What would you like to gain and/or learn from participating in such a support group? Using conceptual analysis, one central theme which emerged was a strong need for support. Parents felt that there was a lack of available support and that the opportunity to avail of peer support would be extremely beneficial. Another theme which emerged was information and education – parents were interested in learning more about young people's mental health as well as DSH statistics, aetiology and treatments. Other themes which surfaced included re-establishing family communication and boundaries, dealing with adolescent discipline issues, and handling further threats or incidents of self harm.

Using information from the Focus Group Meeting, the SPACE (Supporting Parents and Carers) programme was developed. Its content is guided by the needs of the parents and carers and reflects the themes which emerged. It is a group programme which runs over an eight-week time period. Each session involves 10–12 parents meeting with two facilitators, who are experienced mental health professional from the DSH team, for 90 minutes per week. The approach is psycho-educational. Psycho-educational programs are defined as time-limited, closed groups, conducted by health professionals, for the purpose of educating and providing support to its lay membership [13]. Using presentations, video footage modelling effective communication with adolescents, group discussion and exercises the parents are provided with information regarding DSH, support, and the opportunity for communication skills development. The topics for each session are drawn from themes which emerged at the Focus Group Meeting and include the following:

• Information on self harm in young people

• Depression in young people

• Medication – information about medication used in treating depressive disorders in young people

• Parenting adolescents including positive communication, setting boundaries and dealing with emotional and behavioural difficulties that can arise.

• Help with re-establishing family relationships and boundaries after an incident of self harm.

• Advice on how to handle threats or further incidents of self harm

• Self-care – looking after one-self as a parent by achieving balance in life. The importance of parents taking time off to rest and renew themselves.

• Information on resources within the community such as help-lines, counselling services, family support services.

A typical session involves an introduction, feedback from the previous week, introduction of a new topic, small group discussion, feedback to the larger group and a 'thought for the week', where parents are encouraged to consider how the content of the week's programme might be helpful in their interaction with their child.

### Aims of the SPACE programme

The aims of the current study are to evaluate the effectiveness of the SPACE programme in decreasing parental psychological distress as measured by the General Health Questionnaire-12; reducing young peoples' difficulties as measured by the Parental Version of the Strengths and Difficulties Questionnaire; increasing parental satisfaction as measured by the Kansas Parenting Satisfaction Scale and increasing parents' ratings of their own defined challenges and goals as measured by Challenges and Goals Scales.

## Methods

### Study Design

This study used a repeated-measures design to identify significant changes in well-being after treatment. The main independent variable was time, with assessment occurring before (Time 1) and after (Time 2) the SPACE programme. In addition, participants were assessed at a 6-month follow-up session (Time 3). The dependent variables include measures of parent psychological distress, parental satisfaction with their role as parents, parents' ratings of child's difficulties, ratings of challenges and goal achievement.

The study was approved by the Ethics Committee of the Children's University Hospital, Temple Street.

### Participants

Participants were 46 parents of children and adolescents (aged 16 years and under) who were attending mental health services having engaged in or expressed thoughts of DSH. Fourteen child care staff looking after children in residential centres also participated in the study, but as they differed significantly from the parents on several baseline measures, it was concluded that they were two distinct groups, and they have been omitted from the remainder of the study. The resulting sample therefore consisted of 46 parents, of whom 31 (67%) were mothers and 15 (33%) were fathers. They were attending services in relation to 32 young people.

With regard to the number of participants at each time-point – 46 participants completed measures at Time 1. 70% (N = 32) of that original sample completed the measures at Time 2. These numbers decreased again at Time 3 – 37% (N = 17) of the original sample at Time 1 completed measures at Time 3.

Parents were recruited from Temple Street Children's University Hospital, 24 Child and Adolescent Mental Health Teams (CAMHS) and 10 family support services through-out Dublin. With regard to Temple Street Hospital, letters were sent to all parents/carers of young people who had attended the Accident & Emergency department over a four year period (2004–2007) with self harm or suicidal behaviour informing them about the programme and inviting them to attend. Residential homes, CAMHS and family support services were also informed by letter of the programme and invited to refer parents and carers. Once a parent or carer was referred, they were contacted by a researcher and invited to attend the SPACE programme. Over the course of the study 64 parents were referred, of whom 46 (72%) subsequently attended the programme.

### Young Person Characteristics

Of the 32 young people whose parents attended SPACE, 8 (25%) were male and 24 (75%) were female, with a mean age of 13.71 years. When referrals were made to the SPACE programme, referrers were asked to detail the type of self harm that the young person engaged in as well as whether the young person had engaged in previous episodes of self harm. When participants were recruited from the hospital, this information was obtained from the DSH database. With regard to method of DSH, overdose was the most common (50%, N = 16) followed by cutting (26.7%, N = 8). 13.3% (N = 4) presented with other types such as attempted hanging or self biting and 10% (N = 3) presented with suicidal ideation only. In one instance the type of self harm was not specified. 20 (62.5%) of the young people had a history of repeated self harm.

The SPACE programme ran over four cycles. Ten participants were from the first cycle, 13 from the second, 11 from the third, and 12 from the fourth.

### Measures

Parents completed the following measures about themselves:

• The General Health Questionnaire (GHQ 12) is a widely used self report screening tool used for the assessment of mental well-being [14]. It is a measure of common mental health problems/domains of depression, anxiety, somatic symptoms, and social withdrawal. It has well established reliability and validity and has been shown to have internal consistency reliability coefficients of 0.82 to 0.86 in most studies [14,15].

• The Kansas Parenting Satisfaction Scale (KPS) is a 3 item self report measure designed to assess parent-satisfaction with themselves as a parent, satisfaction with the behaviour of their children and satisfaction with their relationship with their children. The scale is reported to have good concurrent validity – significant correlations have been found with the Kansas Marital Satisfaction Scale and the Rosenberg Self Esteem Scale (0.23 to 0.55) [16].

• In addition, parents also completed Challenges and Goals Scales [17] which required them to identify and rate their challenges and goals at the present time.

Parents completed the following measure about their children.

• The Strengths and Difficulties Questionnaire (SDQ) [18] is a brief behavioural screening questionnaire for 3 to 16 year olds. It consists of 5 subscales – emotional symptoms, conduct problems, hyperactivity, peer relationship problems and pro-social behaviour. All subscales except the pro-social behaviour subscale are added together to generate a total difficulties score. The SDQ subscales have a mean internal consistency reliability co-efficient of 0.71, mean test retest reliability co-efficient over 6 months of 0.62 and demonstrates good criterion validity for predicting psychological disorders [19].

### Procedures

At the first session of the programme, parents provided informed consent and completed the survey instruments. At the last session the same measures were administered again. Six months later parents were contacted and asked to attend a booster session, at the start of which they completed the measures for the final time.

### Planned Analyses

In order to identify any significant change in the dependent variables over time, a series of one-way repeated Analysis of Variance tests (ANOVA) were used and alpha was set at .05. Where significant differences were found paired sample t-tests were used to further examine where the differences were. Effect sizes were calculated using Cohen's d. The sample size varied slightly across the analyses due to instances of missing data. In order to account for this, intention to treat analysis was also conducted. Using this analysis it was assumed that no change occurred across time for participants who did not complete measures at one or all data collection points.

## Results

As is common in longitudinal research, this study was the subject to the effects of attrition. While there were 46 participants at Time 1, this reduced to 32 at Time 2 and 17 at Time 3. In order to evaluate the effects of attrition, a series of independent samples t-tests were conducted to compare scores on each measure between those who completed measures at Time 1 only with those who completed measures at Time 1 and Time 2 as well as those who completed measures at all three data collection points. There was no significant differences between these groups on any measures apart from Rating of Challenges where there was a significant difference (t (58) = 2.58, p = .012) between those who completed Time 1 and Time 2 (M = 12.97) and those who completed Time 1 only (M = 9.94). Parents who completed Time 1 and Time 2 had higher ratings of their challenges than those who completed Time 1 only.

### General Health Questionnaire

Before the SPACE programme, mean parental scores on the General Health Questionnaire 12 (GHQ-12) (M = 6.33) were in the caseness range for psychological distress (a score of 3 or above). This had fallen significantly by the end of the programme (M = 3.16) with a further fall to within the normal range (M = 0.88) by Time 3 (Table 1).

**Table 1:** Parental Scores on the General Health Questionnaire 12 before SPACE, after SPACE and at 6-month follow up

**Time**	**Mean**	**SD**	**N**
1	6.33	4.1	45
2	3.16	3.9	32
3	0.88	2.47	17

A one-way repeated measures ANOVA was used to identify any significant difference over time. A significant difference was found (F(2,12) = 34.8, p = .000). Intention to treat analysis was also conducted whereby missing data was replaced with the means for Time1 and a significant difference was found (F(2, 44) = 13.92, p = .000).

A series of paired sample t-tests were then conducted in order to identify where the differences were. Given that multiple t-tests were used, Bonferroni correction for multiple comparisons was made whereby the alpha level was divided by the number of comparisons. Using this more conservative alpha level of 0.017, the means for Time 2 were significantly lower than the means for Time 1 with Cohen's d of 1.39 indicating a large effect size [20]. The 95% confidence interval on the difference between means was 1.89 – 7.68. Furthermore the means for Time 3 were significantly lower than the means for Time 1(95% CI: 4.99 – 8.87) with Cohen's d = 2.79 indicating a very large effect.

The relationship between child and parent factors was investigated using a series of Mann Whitney U Tests. When referrals were made to the SPACE programme, referrers were asked to document whether the child had a previous history of repeated self harm or whether this was a first instance. Cases which had presented to Temple Street hospital only were checked on the DSH database which also documents whether there have been previous episodes of DSH or not. There was a significant association between parental psychological distress at baseline (as measured by the GHQ-12) and previous history of repeated self harm in the child (Z = -2.23, n = 32, p = .026) with high levels of psychological distress associated with previous episodes of DSH.

### Strengths and Difficulties Questionnaire

The Strengths and Difficulties Questionnaire (SDQ) was completed by each parent about their child. With regard to child's Total Difficulties, the mean score was in the abnormal range (17–40) at the beginning of the programme and had decreased into the borderline range (14–16) at the 6 month follow-up session. For each subscale of the SDQ (pro-social behaviour, hyperactivity, conduct problems, emotional difficulties, peer problems), as well as the Total Difficulties score, means and standard deviations for the overall group were calculated and are given in Table 2.

**Table 2:** Parental scores on the Strengths and Difficulties Questionnaire before SPACE, after SPACE and at 6 month follow up

	**Time 1**			**Time 2**			**Time 3**		
	*Mean*	*SD*	*N*	*Mean*	*SD*	*N*	*Mean*	*SD*	*N*
**Total Difficulties**	20.15	5.19	46	17.41	6.31	32	15.06	8.36	17
**Pro-Social Behaviour**	6.09	2.3	46	6.38	2.67	32	6.2	3.22	17
**Hyperactivity**	5.8	2.19	46	5.44	2.42	32	4.71	2.73	17
**Conduct Problems**	4	2.07	46	3.69	2.15	32	2.76	2.44	17
**Emotional Difficulties**	6.93	1.89	46	5.63	2.86	32	4.88	2.96	17
**Peer Problems**	3.41	2.15	46	2.66	2.35	32	2.53	2.24	17

The initial analysis focused on change over time and a series of one-way repeated measures ANOVAs were used to identify any significant differences. A significant difference was found for the Total Difficulties Scale (F(2,12) = 11.827, p = 0.001). Using paired samples t-tests, it was found that the means for Time 2 and Time 3 were significantly lower than the means for Time 1. This difference remained significant using Intention to Treat analysis.

A significant difference was also found for the Hyperactivity subscale (F(2,12) = 4.289 p = 0.039) and the Emotional Difficulties subscale (F(2, 12) = 10.264, p = 0.003.

### Kansas Parenting Satisfaction Scale

Parental satisfaction increased across the three time periods. Means and standard deviations for the overall group on the Kansas Parenting Scale (KPS) were calculated and are presented in Table 3.

**Table 3:** Parental scores on the Kansas Parenting Satisfaction Scale before SPACE, after SPACE and at 6 month follow up

**Time**	**Mean**	**SD**	**N**
1	12.13	3.21	45
2	13.48	3.48	31
3	15	3.48	17

A one way repeated ANOVA was used to identify any significant difference over time. A significant difference was found (F(2,12) = 30.01, p = .000). The means for Time 3 were significantly higher than the means for Time 1 and Time 2. Using Intention to Treat analysis, these statistically significant results remained.

### Challenges & Goals Scale

Parents' ratings of their challenges decreased across the three time periods. Means and standard deviations for the overall group on the Challenges and Goals scale were calculated and are displayed in Table 4.

**Table 4:** Parental ratings of Challenges & Goals before SPACE, after SPACE and 6 months following SPACE

		**Time 1**			**Time 2**			**Time 3**		
		*Mean*	*SD*	*N*	*Mean*	*SD*	*N*	*Mean*	*SD*	*N*
**Challenges**		12.76	3.84	46	8.88	3.95	24	6.65	3.82	13
**Goals**		6.14	2.93	46	10.66	4.18	22	11.35	4.65	13

With regard to parents ratings of their challenges, a one way repeated ANOVA revealed that there was a significant difference over time (F(2,5) = 13.68, p = 0.009). The means for Time 1 were significantly higher than the means for Time 2 and Time 3. Parents' ratings of their goals also increased across the three time periods. A one way repeated ANOVA revealed that there was a significant difference over time (F (2,5) = 6.003, p = .047). The means for Time 2 and Time 3 were significantly higher than the means for Time 1. These results remained significant using Intention to Treat analysis.

## Discussion

This article describes a pilot study evaluating the SPACE programme, a group programme designed to support parents and carers of children and adolescents with Deliberate Self Harm. The study indicates positive results for the parents who completed the programme. Parents had lower levels of psychological distress, higher levels of parental satisfaction, lower ratings on their own defined challenges, and higher ratings of their goals after the programme, and these gains were maintained 6 months after the programme. Parents also reported that their young people had lower levels of total difficulties, hyperactivity and emotional problems following their parent's attendance of the programme. When the principle of Intention to Treat was applied, results remained statistically significant. The fact that this more conservative approach yielded significance only serves to heighten the impact of these findings.

Of particular note is the high number of parents who met the criteria for psychological distress (76%) at Time 1. While this level of psychological distress may reflect parents' reaction to their child's self-harm, it is also possible that it reflects underlying parental psychiatric disorder and psychological distress, which have been shown to be common in families of suicidal young people [21,22]. Either way this psychological distress is likely to be perpetuated by the lack of support available to parents and carers of young people with DSH. It also emphasizes the importance of developing a programme such as SPACE to provide this much needed support to parents and carers and help to alleviate such feelings. Furthermore, parents whose child had a previous history of self harm were found to have higher levels of psychological distress so perhaps the SPACE programme would be particularly beneficial to these individuals.

With regard to the profile of DSH in the 32 young people involved in the study, the male to female ratio reflects that of a large survey of Irish adolescents [1], whereby females were three times more likely to harm themselves than males. In the present study, overdose was the most common method of DSH. This is in contrast to the Irish survey where cutting was the most frequently used method. However this discrepancy is consistent with previous studies whereby cutting was found to be more prevalent amongst a community sample of adolescents [2], while rates of overdosing have been found to be higher in a clinical sample [23].

The findings of the current study suggest that the SPACE programme may be beneficial to parents. However the study was subject to limitations and further evaluation is required in order to conclude this. The study has three significant limitations; the lack of a control group, the lack of information about other interventions which the families may have been receiving, and the high attrition rate. In order to conclude that the SPACE programme is effective it would be necessary to compare it with a group of parents receiving no treatment and/or a group who receive a different treatment. Participants would need to be randomly assigned to conditions in order to ensure that any group biases are evenly distributed. Future research should attempt to identify and quantify any other treatment or support that parents receive during the course of the SPACE programme and subsequently control for this. Such a study is currently planned.

As with most longitudinal research carried out in 'real world' clinical settings, the current study was subject to the effects of attrition. Analyses indicated that those who only completed measures at Time 1 did not differ significantly from other participants on any measures other than Rating of Challenges. Those who completed measures at Time 1 and Time 2 had higher ratings of their own defined challenges compared with those who completed measures at Time 1 only. It is possible that that those who perceived their challenges as greater felt that the group was an important support to them and so continued attending in order to help them to overcome their difficulties. By applying an 'intention to treat' analysis which also showed significant results, we have attempted to address the difficulty presented by the high attrition rate. It may be beneficial for future evaluation studies to adopt a preventive approach to attrition. One possibility would be to obtain a facilitator's rating of parent participation in the group. This data would be used to form a profile of parents who drop out and could be used to alert facilitators to potential 'drop outs' through observation of the group [24].

The participants in this study were parents whose child had been referred to a specialist service or who had presented to an Accident and Emergency department. This makes it difficult to generalize the findings to other parents whose children engage in DSH. Considering that only a minority of adolescents (11.3%) attend medical services following DSH, our sample may represent a small proportion of parents of young people with DSH, and may not relate to parents of young people with DSH which does not come to medical attention.

It is anticipated that these limitations will be addressed in a future study which is being developed. This will involve a randomized controlled trial in which the SPACE programme will be extended to the wider community and will be available to parents who are not in contact with services as well as those who are. Such a study will be able to ensure the generalisability of findings and will address questions regarding comparisons between families who attend health services following DSH with those who do not. Considering that a need identified at the Focus Group Meeting was that of re-establishing communication, the randomized controlled trial will include the communication subscale of the McMaster Family Assessment which incorporates items pertaining to communication within the family [25].

## Conclusion

In conclusion, these findings suggest that the SPACE programme may be an effective means of support for parents of young people with Deliberate Self Harm. Parents who completed the programme experienced positive gains afterwards which were maintained 6 months later. Future research will aim to address some of the limitations associated with the present study such as the ability to generalise from this sample, the lack of a control group, the small sample size and high attrition rate. However these preliminary results suggest that SPACE is a promising development in providing support for parents of young people with Deliberate Self Harm.

## Competing interests

The authors declare that they have no competing interests.

## Authors' contributions

LP was involved in the data collection, statistical analysis and drafted the manuscript. SM and SB participated in the design of the study, data collection and statistical analysis. CB, SC, AC and CF ran the SPACE groups. CF was involved in the conception of the study, participated in its supervision, design and co-ordination as well as critically revising the final draft of the manuscript. SG participated in the design of the study, statistical analysis and interpretation of data. All authors have read and approved the final manuscript.
